# Local Microtubule and F-Actin Distributions Fully Constrain the Spatial Geometry of *Drosophila* Sensory Dendritic Arbors

**DOI:** 10.3390/ijms24076741

**Published:** 2023-04-04

**Authors:** Sumit Nanda, Shatabdi Bhattacharjee, Daniel N. Cox, Giorgio A. Ascoli

**Affiliations:** 1 Center for Neural Informatics, Structures, and Plasticity and Neuroscience Program, Krasnow Institute for Advanced Study, George Mason University, Fairfax, VA 22030, USA; snanda2@gmu.edu; 2 Neuroscience Institute, Georgia State University, Atlanta, GA 30303, USA; sbhattacharjee2@gsu.edu (S.B.); dcox18@gsu.edu (D.N.C.); 3 Bioengineering Department, College of Engineering and Computing, George Mason University, Fairfax, VA 22032, USA

**Keywords:** neuron morphology, *Drosophila* larva, dendritic arborization neurons, microtubule, actin filament, computational modelling, branch angle, branch straightness, arbor geometry, mutated morphology

## Abstract

Dendritic morphology underlies the source and processing of neuronal signal inputs. Morphology can be broadly described by two types of geometric characteristics. The first is dendrogram topology, defined by the length and frequency of the arbor branches; the second is spatial embedding, mainly determined by branch angles and straightness. We have previously demonstrated that microtubules and actin filaments are associated with arbor elongation and branching, fully constraining dendrogram topology. Here, we relate the local distribution of these two primary cytoskeletal components with dendritic spatial embedding. We first reconstruct and analyze 167 sensory neurons from the *Drosophila* larva encompassing multiple cell classes and genotypes. We observe that branches with a higher microtubule concentration tend to deviate less from the direction of their parent branch across all neuron types. Higher microtubule branches are also overall straighter. F-actin displays a similar effect on angular deviation and branch straightness, but not as consistently across all neuron types as microtubule. These observations raise the question as to whether the associations between cytoskeletal distributions and arbor geometry are sufficient constraints to reproduce type-specific dendritic architecture. Therefore, we create a computational model of dendritic morphology purely constrained by the cytoskeletal composition measured from real neurons. The model quantitatively captures both spatial embedding and dendrogram topology across all tested neuron groups. These results suggest a common developmental mechanism regulating diverse morphologies, where the local cytoskeletal distribution can fully specify the overall emergent geometry of dendritic arbors.

## 1. Introduction

Nervous systems comprise a set of distinct neuron types with specific functional roles. During development, each neuron matures into a type-specific axonal–dendritic architecture through a combination of genetic encoding and external influences, such as molecular guidance cues. The overall geometry of the dendritic arbor forms the basis of the neuron’s connectivity and computational properties [1,2,3,4]. The extensive influence of the dendritic structure over neural function is further revealed by the experimentally observed correlations between altered neural morphology and various neurological disorders [5,6,7].

The morphology of dendritic arbors can be conceptualized in terms of two distinct geometric aspects: dendrogram topology and spatial embedding [8]. Dendrogram topology defines tree size (total length) and complexity (number of branches), and affects the biophysical computations carried out by dendrites [9]. Spatial embedding determines the overall shape of dendritic arbors and influences their connectivity to either sensory or axonal inputs [10,11]. Both topological and spatial aspects are exquisitely regulated to optimize each neuron type-specific functional role. 

Mature neuronal arbors emerge from a series of causal events such as growth, retraction, and branching [12,13]. Since each event occurs in three-dimensional space, the orientations of the newly formed branches are also determined simultaneously. Hence, elucidating the biochemical mechanisms underlying these processes entails relating spatially oriented topological events with the mediator molecules that drive arbor growth and plasticity. 

All regulatory processes that control dendritic development, such as cell-specific genetic information, extracellular cues, spatial constraints, and neural activity [1,3], eventually converge on the cytoskeletal mediator molecules, namely actin filaments (F-actin) and microtubules [14,15,16,17,18,19]. Additionally, the modification and maintenance of type-specific neuronal morphology is greatly facilitated by the dynamic self-organization of microtubules and F-actin [20,21]. Cell-intrinsic signaling and combinatorial interactions of transcription factors mold the type-specific dendritic diversity by regulating cytoskeletal composition [22,23,24,25]. While numerous upstream signaling pathways that regulate both microtubules and F-actin dynamics have been described [15,23,26,27], the specific roles of microtubules and F-actin in the causal steps that produce dendritic morphology are only partially understood. Many molecular pathways ultimately converge on microtubules and F-actin. Thus, it is important to directly quantify the influence of these cytoskeletal constituents on dendritic architecture across distinct neuron types. 

In the current study, we identify and quantify the relations of microtubules and F-actin distributions with overall dendritic geometry. We also evaluate whether these measured associations remain consistent across a broad range of wild-type and mutated neuron classes representing a wide variety of size and complexity. We finally employ computational simulations to ascertain whether these measured associations are sufficient to describe overall dendritic structure. Specifically, we build a computer model to generate virtual dendritic trees using the microtubules and F-actin distributions measured from real neurons as the sole constraints. 

*Drosophila* sensory multidendritic dendritic arborization (da) neurons are an excellent experimental system for studying dendritic development [14,26]. The optimal location of their dendritic arbors below the translucent larval surface allows for accurate in vivo imaging. The availability of a myriad of genetic tools in the fly system facilitates investigations that can link molecules to morphology. Out of the four classes of da neurons, Class I and Class IV neurons represent the two extremes of the morphological spectrum. While Class I neurons are the smallest and simplest, Class IV neurons are the largest and most complex. These two sensory neuron types are also functionally distinct. Because of their contrasting structures, we choose these two classes for our study. Specifically, we focus on two Class I subtypes (ddaE and vpda) and on one Class IV subtype (ddaC). Additionally, we include several mutated neuron types to check whether the influence of microtubules and F-actin over morphology changes when their arbor-wide distributions are altered through genetic manipulation. 

Previously, we demonstrated that, in sensory neurons from the *Drosophila* larva, microtubule concentration is correlated with downstream arbor length, while actin filaments are enriched at branch points. We also reported that an arbor generation model can accurately reproduce dendrogram topology by controlling key events such as elongation, branching, and termination solely based on local cytoskeletal composition [19]. While dendrogram topology is an essential component of neural structures, the spatial embedding of dendrites is of great importance as well [28]. Two trees with the same topology can potentially emerge with vastly distinct overall shapes if their branch angles and straightness differ. This study directly investigates the relations of microtubules and F-actin with branch angles and branch straightness, the two parameters that determine the spatial embedding of dendritic arbors.

We reconstructed mature Class IV and Class I dendritic arborization neuron morphologies from confocal image stacks containing arbor-wide signals for microtubules and F-actin quantity [15,23,29]. We then evaluate whether the quantities of these two major cytoskeletal components correlate with measures of dendritic arbor spatial embedding. We observe that a higher quantity of local microtubules is associated with reduced angular deviations and straighter branches. We also notice significant effects of F-actin, albeit not as consistent as those of the microtubules. Informed by these correlations, we proceed to test whether local cytoskeletal composition alone is sufficient to reproduce all emergent geometric features of dendritic morphology, including spatial embedding. Hence, we create a computational model where the microtubules and F-actin distributions determine not only branch elongation, bifurcations, and terminations, but also the straightness and local orientation of all branches.

## 2. Results

We studied 167 *Drosophila melanogaster* larval da neurons from 17 distinct groups, including wild-type (WT) controls and mutants in the simplest Class I and the most complex Class IV neurons. Four Class I groups included two WT subtypes (ddaE and vpda) and two mutants of the vpda subtype (*mts* knockdown and *mts* overexpression). Thirteen Class IV groups, all of subtype ddaC, included WT and twelve mutants: *bdwf* knockdown, *form3* overexpression, *mts* knockdown, *mts* overexpression, *ct* knockdown, *RpL4* knockdown, *RpL17* knockdown, *RpL22* knockdown, *RpL31* knockdown, *RpL35A* knockdown, *RpS10b* knockdown, and *RpS24* knockdown (see Table 1 for the number of neurons in each type). 

### 2.1. Distinct Dendritic Architecture of Sensory Neurons 

**Figure 1:**
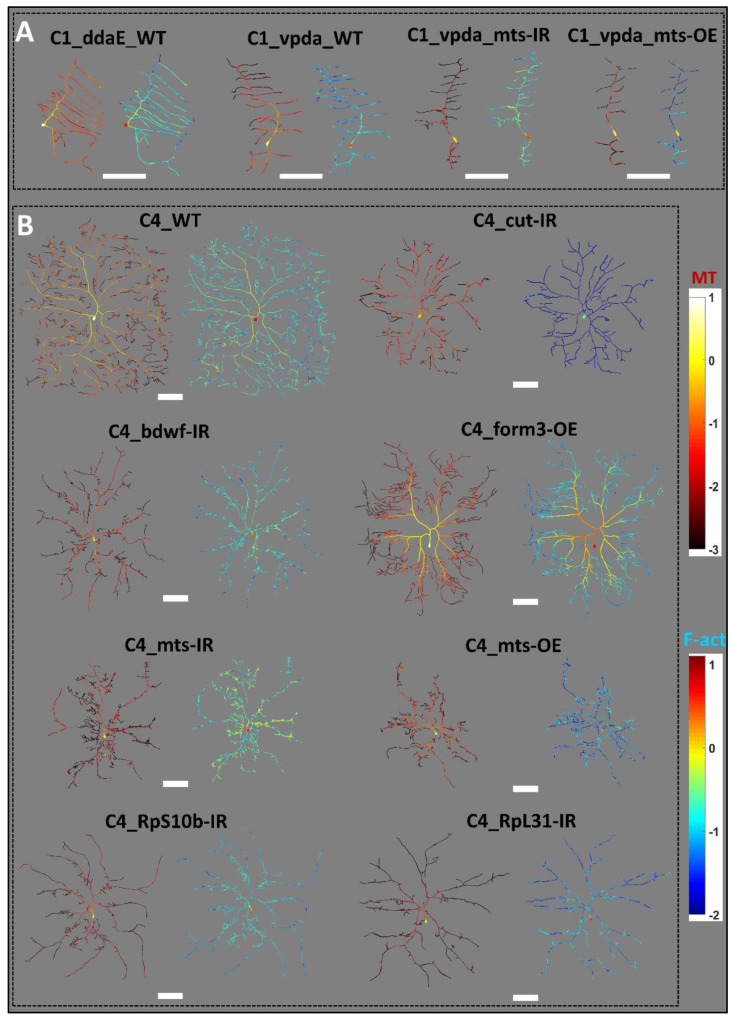
Morphological Diversity of Sensory Neurons in the Drosophila Larva. Single representative neurons from (**A**) 4 Class I neuron types (from left to right: WT ddaE, WT vpda, vpda mts-IR, and vpda mts-OE) and (**B**) 8 Class IV neuron types (from left to right, then top to bottom: WT, cut-IR, bdwf-IR, form3-OE, mts-IR, mts-OE, RpS10b-IR, and RpL31-IR). Each neuron is shown in two separate images side by side, displaying microtubule (left, red hue) and F-actin (right, green-blue hue) distributions, respectively. Color scales on the right side of the figure, constant for all 17 neuron groups, represent the log_10_ of microtubule (right-top) and the log_10_ of F-actin (right-bottom) quantities, respectively. Scale bars below each single neuron image-pair correspond to 100 µm.

The morphological appearance differed substantially between Class I and Class IV neurons as well as among genotypes. These differences in overall morphology were accompanied by changed microtubule and F-actin distributions across the dendritic arbors (Figure 1). Class I ddaE and vpda subtypes also have distinctly different shapes. The ddaE neurons have comb-like secondary branches extending towards the same direction from the primary branch, whereas the vpda secondary branches extend towards both sides. All Class IV ddaC mutants invade a reduced spatial surrounding compared to the WT control ddaC neurons. This suggests that, in all the genetic perturbations we analyzed for Class IV neurons, irrespective of whether knockout or overexpression, the phenotypic defect leads to a less optimized space-filling architecture (Figure 1). While the distribution of microtubule and F-actin varied across neuron types, there are also persistent patterns in their distribution. Both microtubules and F-actin are in higher quantity closer to the cell body. Microtubule density tends to decrease gradually while traversing across the arbors in the anterograde direction. F-actin quantity, while also demonstrating a general reduction with the distance from the soma, demonstrated greater variability across the branches. Most terminal branches were low in microtubule quantity in all neuron types, but a substantial proportion of them expressed a higher F-actin quantity (see Figure 1 for differences in MT and F-actin distributions). 

We observe a distinctive morphological signature in the distributions of branch lengths and angles of *Drosophila* sensory neurons. Specifically, in *Drosophila* Class IV neurons, terminal branches are significantly (*p* < 0.05) shorter (12.9 ± 12.3 µm) than internal branches (17.6 ± 15.3 µm). Moreover, branch angles increase with branch order in both class I ddaE WT (binned correlation R = 0.77, *p* < 0.05) and Class IV ddaC WT (binned correlation R = 0.2, *p* < 0.05) neurons (see Table 1 for number of neurons in each group; see Supplementary Materials Table S1 for correlation values of all neuron groups). Interestingly, these architectural features are opposite to those reported in other neural systems (see the Discussion section).

### 2.2. Influence of Local Cytoskeleton Composition on Branch Orientation and Straightness 

**Figure 2:**
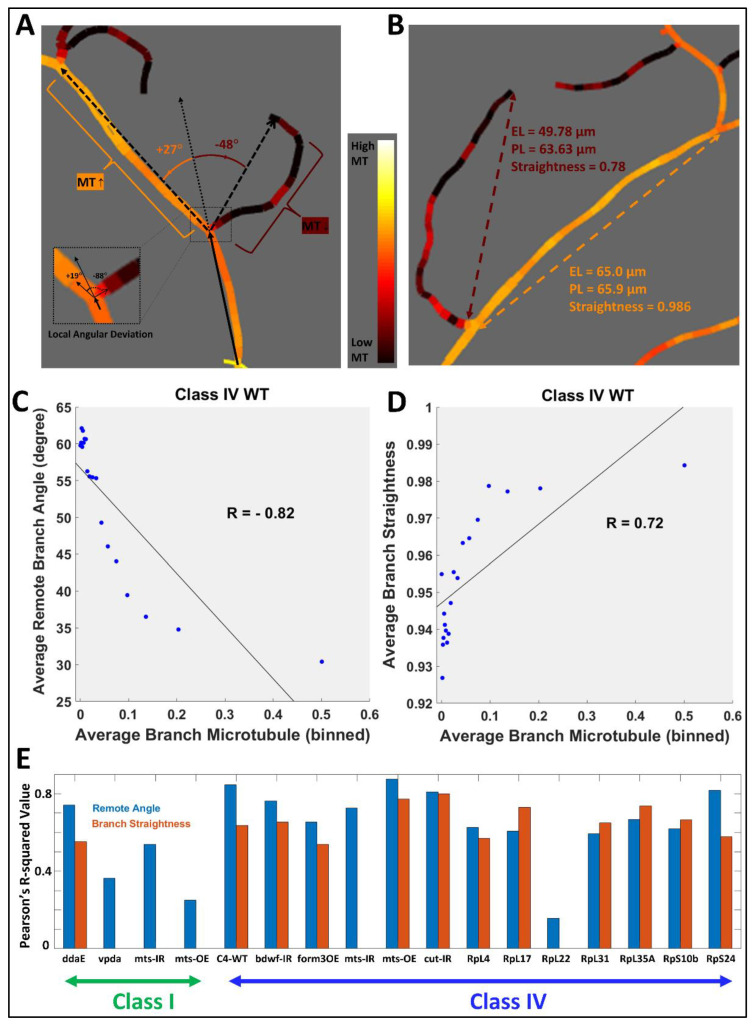
Branch Angular Deviation and Straightness. (**A**) Schematic illustration of remote angular deviation (inset: local angular deviation) in counterclockwise (positive sign) and clockwise (negative sign) direction. The branches with lower average microtubule have greater angular deviations. (**B**) Schematic illustration of branch straightness (ratio of branch Euclidean length to branch path length). The branches with higher average microtubule are straighter. (**C**) Binned scatter plot of average remote branch angle against average branch microtubule quantity of Class IV WT neurons demonstrates a negative correlation. (**D**) Binned scatter plot of average branch straightness against average branch MT quantity of Class IV WT neurons demonstrates a positive correlation. (**E**) Coefficients of determination of branch MT with branch remote angle (blue) and straightness (orange). Class I vpda WT and mutants show no correlation between MT quantity and branch straightness. See Table 1 for sample size by cell type.

In order to estimate the relative influence of cytoskeletal composition over spatial geometry, we measured the angular deviation (Figure 2A) and straightness (Figure 2B) of every branch in each neuron across all groups and computed their correlations with MT and F-actin quantities (see the Materials and Methods section for details). In all 17 neuron groups, the remote branch angle was strongly anti-correlated to the average branch MT quantity (Figure 2C and Table 2). Moreover, 12 out of 17 neuron groups, including Class IV ddaC WT and Class I ddaE WT, demonstrated a positive correlation between branch straightness and average branch MT quantity (Figure 2D and Table 2). This suggests that local microtubules not only influence the overall orientation of a dendritic branch relative to its parent branch, but also the extent of its straightness. For Class I vpda neuron groups (WT, *mts-IR*, and *mts-OE*), the only significant correlations were between average branch microtubule and remote branch angle (Figure 2E). This could partly be explained by the fact that Class I vpda neuron branches are largely straight and lack much variability that could be explained by differential cytoskeletal distributions. 

Average branch F-actin quantity was also negatively correlated with remote branch angle in Class I ddaE WT neurons as well as in several Class IV ddaC genotypes, namely WT, *form3-OE*, *mts-IR*, *RpS10b-IR,* and *RpS24-IR* (Table 2). In contrast, in Class IV *cut-IR* neurons, average F-actin was positively correlated with remote branch angle. This reverse effect is likely due to the extremely low levels of F-actin in *cut-IR* dendritic arbors compared to other neuron types. Class IV *cut-IR* has the minimum average F-actin level amongst all the studied neuron types, merely 8% of Class IV WT F-actin level and less than a third of the next lowest, i.e., Class IV *mts-OE* (see Figure 1 for F-actin distribution of *cut-IR*). The correlations between remote branch angle and F-actin were not statistically significant in the remaining groups. At the same time, average branch F-actin was positively correlated (such as the average microtubule) with branch straightness in 11 out of the 17 neurons groups, with the remaining ones, including all Class I groups, showing no significant correlation. Branch angle and straightness had stronger correlations with F-actin than with MT quantity only in the case of Class IV *formin 3* overexpression. 

Similar to local microtubules, integral microtubules (see the Materials and Methods section for definition) was also negatively correlated with the remote branch angle in 6 out of the 17 neuron groups (the remaining groups demonstrated no correlation) and was positively correlated with branch straightness in 11 out of 17 groups (Table 2). 

### 2.3. Relation between Angular Deviations of Sibling Branches

**Figure 3:**
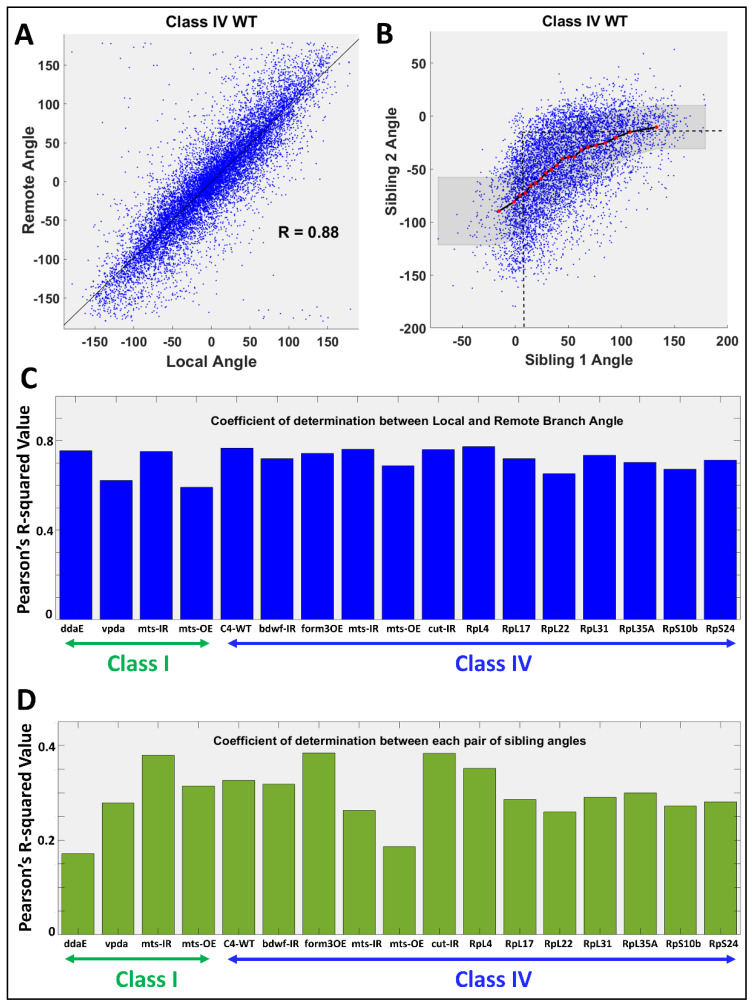
Analysis of Branch Angular Deviation. (**A**) Scatter plot of remote vs. local branch angles reveals strong positive correlation in Class IV WT neurons. (**B**) The signed angular deviations of sibling branches (also in Class IV WT neurons) are positively correlated and they usually deviate in opposite directions. This corresponds to a negative correlation between the absolute angular deviations (see Supplementary Materials Table S2). (**C**) Coefficients of determination between local and remote branch angles across the 17 neuron groups. (**D**) Coefficients of determination of the signed remote angle between each pair of sibling branches across the 17 neuron groups.

Local and remote branch angles were highly correlated across all neuron groups (Figure 3 and Table S2). This result demonstrates that angular orientations attained during branch formation tend to remain substantially preserved until the end of the branch (Figure 3A,C). When we compare the sibling branch pairs, we also observe that the larger of the two sibling angles is highly correlated with the branch tilt (R = 0.88 for Class I ddaE WT, R = 0.94 for Class I vpda WT, R = 0.94 for Class IV ddaC WT, see Supplementary Materials Table S2 for the remaining neuron groups). In most cases, moreover, the pairs have opposite signs of angular deviation, i.e., if one sibling deviates clockwise relative to the parent branch vector, the other would tend to deviate counterclockwise. Furthermore, the signed angular deviations of the two sibling branches are positively correlated: when the counterclockwise branch deviates more from the parent’s direction (i.e., increasing in the positive direction), the clockwise branch will deviate less (decreasing in the negative direction towards 0), and vice versa (see Figure 3B,D). Not surprisingly, when we remove the signs and observe the correlations between the absolute angular deviations of each branch sibling pair, we observe a negative correlation across all neuron types (Supplementary Materials Table S2). This “angular homeostasis” expands the repertoire of morphological homeostasis phenomena previously observed both in *Drosophila* [13] and in the mammalian brain [32]. 

The above observations complement previous results implicating local MT and F-actin quantities in the quantitative specification of dendrogram topology [19]. These measured associations remain broadly consistent across all neuron types. All correlations between branch angle and cytoskeleton (microtubule, F-actin, and integral microtubule) were either negative or non-significant (except with F-act in *cut-IR*, which has highly reduced F-actin). All correlations between branch straightness and cytoskeleton were also either positive or non-significant. We further observe a homeostatic interplay between each branch sibling pair, again, consistent across all neuron types. 

Combined with the previously reported correlation between cytoskeleton and topology [19], are the measured associations of cytoskeletal composition with branch angles and straightness sufficient to fully reproduce the complete arbor geometry of da neurons from various groups? To answer this question, we build a tree generation model where all aspects of arbor geometry, including branching probability, downstream arbor length, branch orientation, and branch straightness are constrained solely by arbor-wide microtubule and F-actin distributions measured from real neuronal arbors. 

### 2.4. A Cytoskeleton-Driven Generative Model of Dendritic Morphology

**Figure 4:**
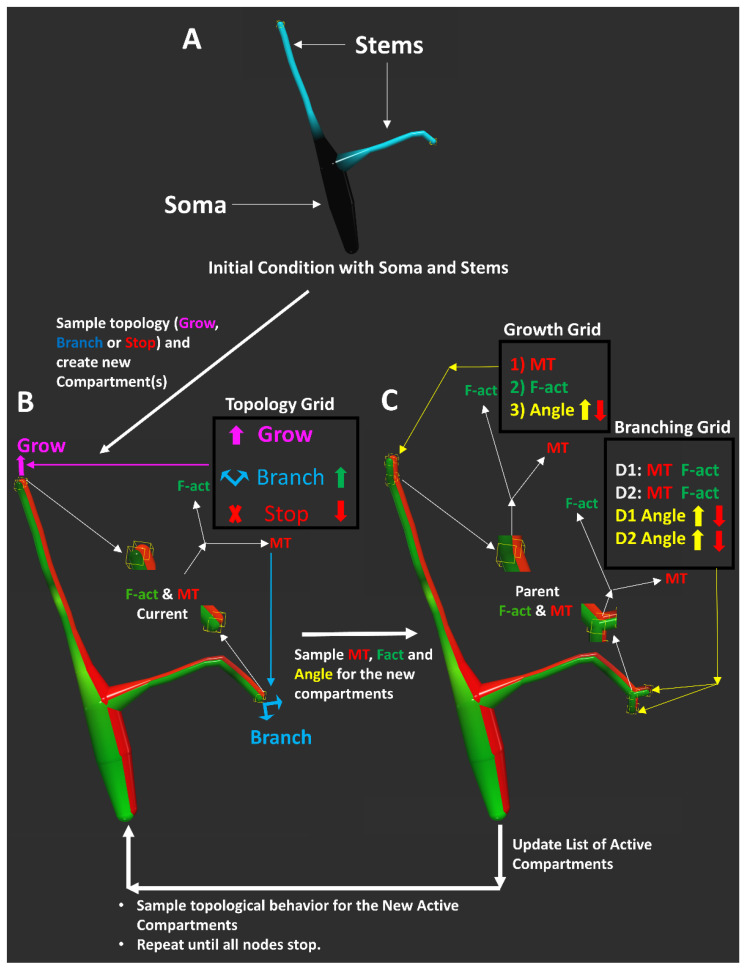
Computational Model of Dendritic Morphology. (**A**) The tree generation process starts with the initial MT and F-actin concentration of the soma and the dendritic stem end points as active compartments. (**B**) For each active compartment, the event sampling determines whether to grow (adding one new compartment), branch (adding two new compartments), or stop (ending the branch). Enriched F-actin (upward green arrow) increases branching probability. Reduced microtubules (downward red arrow) increase termination (Stop) probability. (**C**) If a node grows or branches, the cytoskeletal composition of the newly created compartment is sampled from the corresponding data grids along with their angular deviations relative to their parent compartments. Reduced microtubule (downward red arrow) leads to increased angular deviation (upward yellow arrow). The process is repeated for all newly added compartments, until all branches stop.

Every iterative step of the computational model for virtual tree generation consists of the simultaneous addition of one or two compartments at each growing branch (Figure 4). Individual compartments provide the highest resolution of spatial embedding as well as cytoskeletal composition of a digital dendritic arbor. Even branch-level metrics such as straightness reflect an emergent property of the collective orientations of individual compartments. Thus, the compartment-level simulation not only allows for accurate local geometric embedding, but also more closely resembles the developmental process of dendritic trees. Informed by our experimental observations, the addition, cytoskeletal composition, and the 3D embedding of newly generated compartments in the model are constrained by the local MT and F-actin quantities of the previous compartments, as sampled from real neurons (see the Materials and Methods section for additional details of the sampling process). 

The tree generation process starts with the MT and F-actin quantities of the soma and the dendritic stem end points as the active compartments (Figure 4A). At each iteration, two sampling processes are carried out at every active compartment. The first sampling process (detailed in the Materials and Methods section) determines the topological fate (elongation, branching, or termination) of the active compartment based on its MT and F-actin quantities (Figure 4B). During this process, enriched F-actin increases the branching probability, while MT depletion increases the termination probability [19]. The second sampling process determines the cytoskeletal composition and orientation of each newly generated compartment (Figure 4C). Here, a decreased level of MT in the parent compartment leads to greater angular deviation. If a node elongates or bifurcates, the newly created compartments then become the new active compartments, and the sampling process iteration continues until all branches terminate. The same arbor generation model is employed for all 17 neuron groups.

### 2.5. Simulated Neurons Reproduce all Morphological Attributes across Neuron Groups

**Figure 5:**
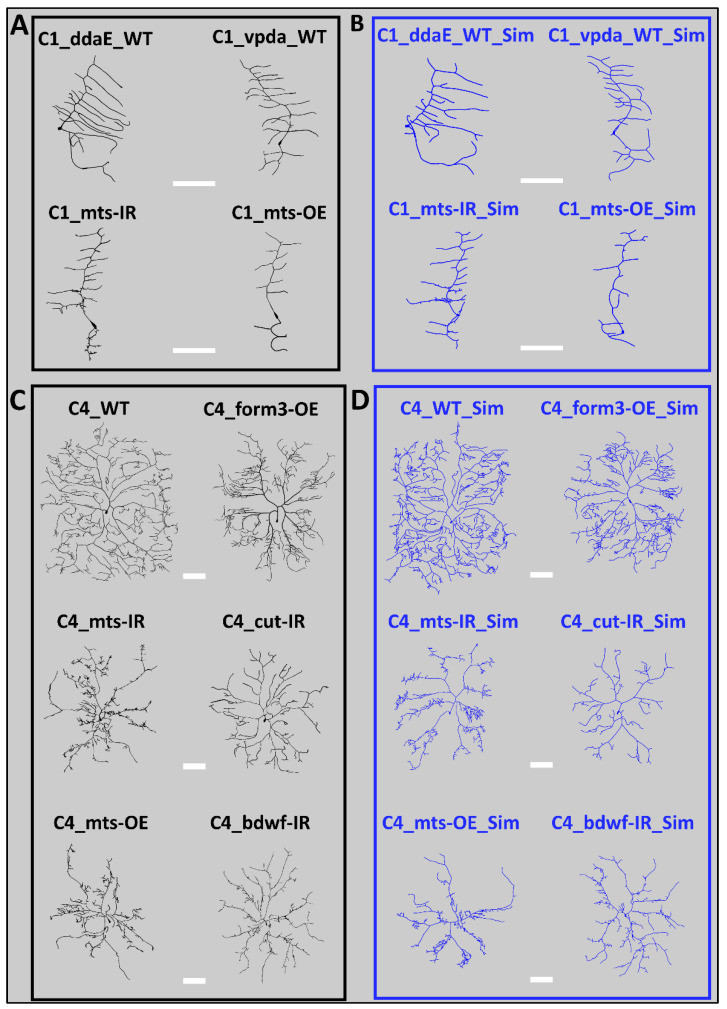
Morphological Comparison Between Real and Simulated Neurons. (**A**) Representative real neurons (black) from the four class I neuron groups (ddaE WT, vpda WT, vpda mts-IR, and vpda mts-OE). (**B**) Representative simulated neurons (blue) from the four class I neuron groups (ddaE WT, vpda WT, vpda mts-IR, and vpda mts-OE). (**C**) Representative real neurons (black) from six Class IV neuron groups (Class IV WT, form3-OE, mts-IR, cut-IR, mts-OE, and bdwf-IR). (**D**) Representative simulated neurons (blue) from six Class IV neuron groups (Class IV WT, form3-OE, mts-IR, cut-IR, mts-OE, and bdwf-IR). All scale bars (white rectangles) represent 100 µm.

To evaluate the effectiveness of the tree generation model, the simulated virtual neurons were first compared visually against their real counterparts. Overall, this qualitative inspection revealed no observable differences in either morphological appearance (Figure 5) or cytoskeletal distribution (Supplementary Materials Figure S1) between real and simulated neurons in any of the cell subclasses, subtypes, and genotypes. 

We then compared numerous quantitative morphological parameters between real and simulated neurons from all 17 neuron groups. We started with the comparison of 10 scalar L-Measure morphometrics properties: (1) number of branches, (2) height, (3) width, (4) total length, (5) maximum path distance, (6) maximum Euclidean distance, (7) maximum branch order, (8) average branch straightness (called “contraction” in L-Measure), (9) average partition asymmetry, and (10) average bifurcation angle. A total of 170 statistical tests (17 neuron groups times 10 L-Measure features) demonstrated no significant difference between real and simulated morphologies (Table 3). 

**Figure 6:**
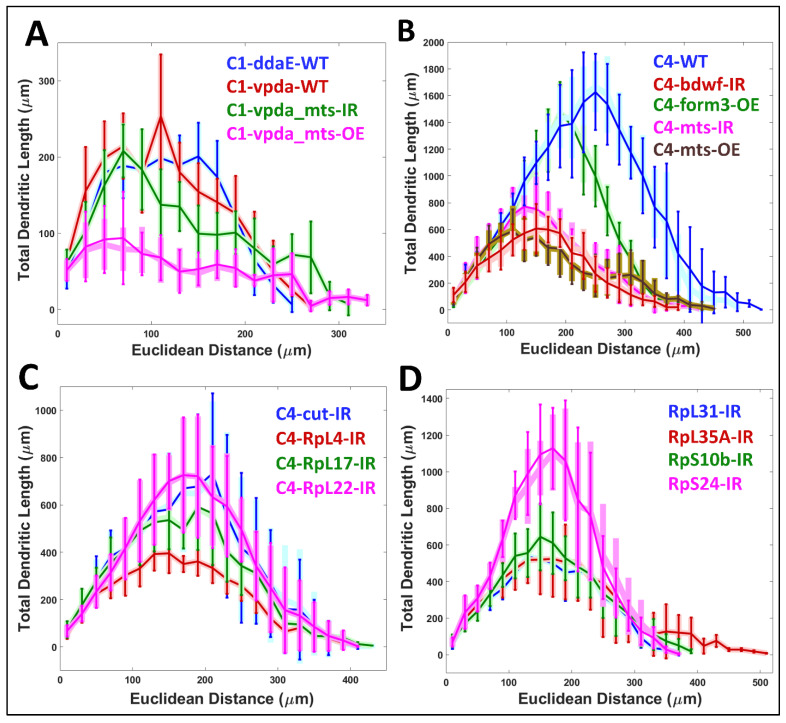
Sholl-Like Comparison Between Real and Simulated Neurons. Sholl-like analysis of total dendritic length against Euclidean distance from the soma for (**A**) Class I and (**B**–**D**) Class IV neurons. Lighter, broader shades of blue, red, green, pink, and brown represent different subclasses and genotypes of the real neurons. Darker, thinner lines in analogous colors represent the corresponding subclasses and genotypes of the simulated neurons.

Next, we compared the Sholl-like distribution of total dendritic length against Euclidean distance between the real and the simulated morphologies, and found no observable difference in any of the neuronal groups (Figure 6). 

To quantify this comparison, we also employed persistence vectors, a representation of arbor morphology that captures branch distributions similarly to Sholl diagrams but enabling the definition of a formal metric distance [33]. If the real and simulated neurons had different branch distributions, the distance between a real and a simulated neuron would be, on average, larger than the distance between two real or two simulated neurons. The persistence vector arccosine distance was thus measured for each pair of neurons within condition (two real neurons or two simulated neurons) or between condition (one real and one simulated neuron). The within- and between-condition pairwise distances were then compared by *t*-test, and no significant difference was found for any of 17 neuron groups (Table 4). 

**Figure 7:**
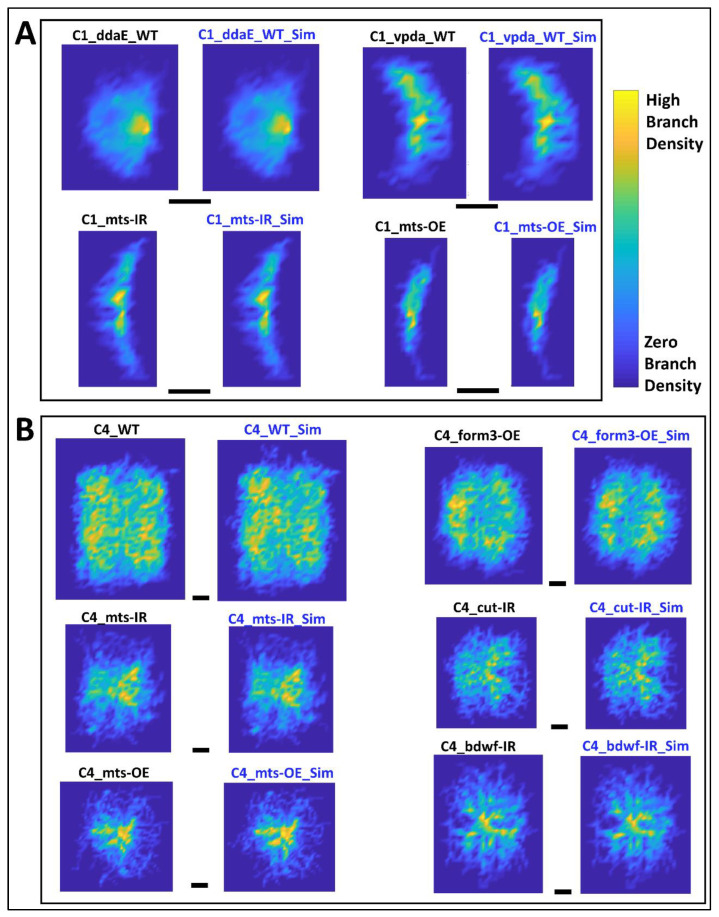
Average Arbor Density of Real and Simulated Neurons. (**A**) Arbor density averaged across all neurons from each group for Class I neuron types (ddaE WT, vpda WT, vpda mts-IR, and vpda mts-OE). Heatmaps for both real (left) and simulated (right) neurons are provided for each group. (**B**) Arbor density averaged across all neurons from each group for six Class IV neuron groups (WT, form3-OE, mts-IR, cut-IR, mts-OE, and bdwf-IR). Black scale bars below each real-simulated neuron pair represent 100 µm. Heatmaps for both real (left) and simulated (right) neurons are provided for each group, where yellow in the color bar represents high branch density and blue in the color bar represents low to zero branch density. The heatmaps for the 7 remaining Class IV neuron groups are included in Supplementary Materials Figure S2.

Lastly, we comparatively analyzed the overall spatial geometry of the real and simulated morphologies by extracting their arbor density matrices, which capture the dendritic density profile of a neuron throughout the invaded region [34]. The average density matrix of each real neuron group was qualitatively similar to that of the corresponding, simulated neuron groups in both Class I and Class IV cells (Figure 7) and for all subtypes and genotypes (Supplementary Materials Figure S2). To quantify these observations, we vectorized the density matrices of each individual neuron and measured all pairwise arccosine distances, similarly to the persistent vector analysis. Within-condition (real–real or simulated–simulated) distances were not statistically different from between-condition (real–simulated) distances (Table 4). 

Collectively, these analyses demonstrate the sufficiency of the cytoskeletal determinants in reproducing all geometric features of Class I and Class IV fruit-fly sensory da neurons under the control and mutant conditions tested. Class I and Class IV neurons have distinct morphological structures. The mutations in these classes lead to even broader morphological diversity. However, the influence of microtubule and F-actin is overall consistent through all tested neuron groups: differential distributions of the two cytoskeletal parameters lead to distinct morphological features in the various studied cell classes and genotypes. 

## 3. Discussion

*Drosophila* sensory da neurons innervating the barrier epidermis of the larva are a versatile model system to study dendritic morphology. While MT and F-actin have been shown to be directly involved in dendritic growth, maintenance, and plasticity [35,36], their precise roles are only recently being elucidated [19]. 

In this study, we quantified neuronal morphology simultaneously with the cytoskeletal distributions from a diverse set of cell classes and mutated phenotypes. The studied mutations included RNAi knockdowns and overexpression of specific genes. The transcription factor *cut* promotes F-actin dynamics by exerting combinatorial regulation through downstream effector molecules [14]. Cut positively regulates *bdwf* expression, which encodes a protein that regulates proportional growth and branching of dendritic arbors [30]. Overexpression of *bdwf* in turn downregulates *cut* expression in da neurons. Both *bdwf-IR* and *bdwf-OE* phenotypes demonstrate branching defects with reduced arbor size and complexity in both Class I and Class IV neuron types. The ribosomal proteins *RpS24*, *RpL4*, *RpL22,* and *RpL31* are physical interactors of *bdwf*. Similar to *bdwf-IR*, knockdown of any of these four proteins leads to a reduction in dendritic complexity, as well as a reduction in average cytoskeletal distributions [30]. Another downstream effector molecule of *cut*-mediated transcriptional regulation is *PP2A* phosphatase. Overexpression and knockdown of PP2A’s catalytic subunit *mts* demonstrates that *PP2A* homeostatically promotes the dendritic development of Class IV and restricts Class I branching, respectively. Both microtubule stability and F-actin organization are regulated by *mts* as well. Knockdown of *mts* leads to reduced complexity in Class IV and increases in short branches in Class I da neurons. Overexpression of *mts* leads to reduced complexity in both Class I and Class IV da neurons [15]. Another regulator of cytoskeletal organization is *formin3* or *form3,* which increases dendritic arbor complexity. *Form3* also promotes maintenance of stable microtubule fibers in the dendrites. RNAi knockdown of *form3* reduces overall dendritic length and complexity. Overexpression of *form3* promotes exuberant terminal dendritic branching, coupled to a proximal shift in branching and cytoskeletal distributions [23]. Knockdown of three additional ribosomal proteins, *RpL17*, *RpL35A,* and *RpS10b*, causes morphological and cytoskeletal defects in da neuron architecture (Figure 1, Figure 6 and Figure 7, and Supplementary Materials Table S1). While all the studied mutations affect the organization, stability, and dynamics of the cytoskeleton, in this study we find that the influence of the cytoskeletal molecules over geometry remains consistent across classes and genetic manipulations. 

Our previous study [19] demonstrated the association between MT and F-actin and the primary causal parameters of dendrogram topology, namely downstream arbor length and branching probability. Specifically, the local microtubule quantity predicts the total dendritic length from that location to all the subsequent terminations. Thus, splitting microtubule resources at branch points partitions the arbor length between the corresponding subtrees. At the same time, F-actin enrichment makes a compartment more likely to bifurcate. These two parameters thus fully determine mature arbor size (total dendritic length) and complexity (number of branches). The present study expands on those prior observations by interrelating the local cytoskeleton composition with the spatial geometry of dendritic arbors in addition to their dendrogram topology.

Previous studies have shown that dendritic orientation is influenced by extracellular guidance cues [37], and transcriptional mechanisms direct primary dendritic orientation towards active axon terminals and away from inactive axons [38]. Prior computational analyses also quantified cell-autonomous relationships between topology and branch angles [39,40]. Earlier models have used path distance from soma or centrifugal branch order to constrain the simulation of realistic dendrogram topology [41]. Here, we identify the influence of cytoskeletal mediator molecules on branch orientation and straightness. Our results indicate that MT quantity acts as a resistance to dendritic tortuosity and to angular deviation from the previous branches: increased MT levels tend to lead to smaller branch angles across all tested morphological classes, subtypes, and genotypes, as well as to straighter branches in most neuron groups. F-actin is also positively correlated with branch straightness in most neuron groups. Similar to microtubule, F-actin demonstrates a negative correlation with branch angle. However, the correlation of F-actin with branch angle is only significant in a minority of cell subtypes and genotypes. Overall, the distribution of both cytoskeletal elements helps dendritic arbors maintain their existing path. 

The arbor generation constrained by MT and F-actin reproduces all geometric properties in both Class I and Class IV da neurons across WT and a variety of genetic mutations. Notably, the computational model regulates the sampling of topological events, cytoskeletal composition, and spatial orientation solely based on MT and F-actin quantity. Class I and Class IV da neurons lie at the opposite ends of the spectrum of arbor complexity among fruit fly larval da neuron subclasses. Moreover, all genetic mutations considered here substantially alter the neuronal arbor relative to their WT counterparts. It is thus noteworthy that the same generative model qualitatively and quantitatively captures the dendritic morphology of all tested neuron groups despite their considerable morphological diversity. We interpret this finding by speculating that, among da neurons, distinct morphological classes and genotypes essentially differ in the cytoskeletal composition but share a common developmental program. 

Previous studies of basal dendrites in neocortical neurons reported a negative correlation of branch angles with centrifugal branch order [28,42]. In contrast, we find that branch angles increase with centrifugal branch order in both Class IV and Class I WT neurons. Moreover, in cortical neurons, both dentate gyrus granule-cell dendrites [43] and basal dendrites of pyramidal cells [42] have longer terminal branches than internal branches, whereas we quantified an opposite phenomenon in fly larval da neurons. These contrasting relations could be attributed to the distinct source of input signals on the dendritic arbors of sensory neurons relative to the basal dendrites of cortical neurons, which receive synaptic contacts from local and long-range axons. It would be interesting to examine the cytoskeletal composition of non-sensory neurons to ascertain if it recapitulates the observed differences in the association between branch angles and centrifugal order. 

The present study demonstrates that cytoskeletal quantity is consistently correlated with spatial geometry across cell types and genetic manipulations. At the same time, it is important to also consider the structural and functional requirements that may also play a role in shaping da neuron arbors. Specifically, these sensory cells receive input from the external environment and their dendritic trees lie flat on the cuticle surface. Nevertheless, the computational simulations further corroborate the measured cytoskeleton–geometry associations by showing that the experimental cytoskeletal distributions are sufficient to fully constrain the emergent arbor morphology of real neurons. 

Despite this ‘proof by construction’, the model cannot by itself guarantee that the cytoskeletal compositions always cause or even precede the formation of the mature arbor: the MT and F-actin quantities as measured in our data might instead have changed after the arbor reached its final shape. However, experimental evidence from electron microscopy is consistent with a lack of MT in terminal branches [44]. Furthermore, F-actin dynamics have been shown to precede branching events [45,46] and synaptogenesis [47]. Recent models have also elucidated the role of F-actin dynamics in Class III da neuron branching [48]. Pinning down the temporal sequence of cytoskeletal changes underlying dendritic growth will ultimately require time-lapse reconstructions of multi-channel image stacks in vivo. That approach involves the meticulous morphological tracing and cytoskeletal quantification from each individual time point, an extremely challenging task at the level of complete arbors [49,50,51].

## 4. Materials and Methods

Multi-signal morphological reconstructions were acquired using previously described methods [29,52], as briefly summarized below. The processed data were deposited in Mendeley.com along with the analysis and modeling code for open-access release upon publication of this manuscript (DOI: 10.17632/cj69j8bpn8.1). The same Mendeley.com package also includes all analysis and simulations scripts, which are released open source to facilitate reproducibility and further community development [53]. The digital tracings and enhanced ESWC files of all 167 neurons were submitted to NeuroMorpho.Org [54] as part of the Ascoli and Cox archives (Table 1). 

### 4.1. Drosophila Strains and Live Confocal Imaging

*Drosophila* stocks were reared at 25 °C on standard cornmeal-molasses-agar media. Age-matched wandering third instar larvae of both sexes were used for all experiments. Fly strains were obtained from Bloomington and the Vienna *Drosophila* Research Center. Fly strains used in the study included: *UAS-GMA::GFP; GAL4[477]*, *UAS-mCherry::Jupiter* [14] (Class IV cytoskeletal reporter strain); *UAS-GMA::GFP;+;GAL4[221]*, *UAS-mCherry::Jupiter* (Class I cytoskeletal reporter strain) [15]; *UAS-form3-B1* [55], *UAS-bdwf-IR* (*B27997*)*, UAS-mts-IR (B57034), UAS-mts (B53709), UAS-ct-IR (B33965), UAS-RpL4-IR (v101346), UAS-RpL17-IR (v105376), UAS-RpL22-IR (v104506), UAS-RpL31-IR (v104467), UAS-RpL35A-IR (v100797), UAS-RpS10b-IR (v106323), UAS-RpS24-IR (v104676)*. *Oregon R* was used as a genetic background control for outcrosses to the multi-fluorescent cytoskeletal transgene reporters. We previously confirmed that expression of the F-actin and microtubules transgene reporters did not themselves exert any effects on dendritic development [14]. Virgin flies from either *UAS-GMA; GAL4[221]*, *UAS-mCherry::JUPITER*, or *UAS-GMA; GAL4[477]*, *UAS-mCherry::Jupiter* were crossed to gene-specific *UAS-RNAi* or *UAS* overexpression (OE) transgenic males. UP-TORR (www.flyrnai.org/up-torr/, accessed on 1 February 2023) was utilized to evaluate predicted off-target effects for gene-specific RNAi transgenes [56]; all RNAi lines used in this study have no predicted off-target effects. Live confocal imaging of mature da neuron Class I and Class IV arbors was performed as previously described [13,14,29]. Briefly, larvae were placed on a glass slide and immersed in a 1:5 (*v*/*v*) diethyl ether to halocarbon oil and covered with a 22 × 50 mm cover slip. 

Neurons expressing fluorescent protein cytoskeletal reporter transgenes were visualized on a Zeiss LSM 780 confocal microscope. Images were collected as z-stacks at a step size of 1.5 microns and 1024 × 1024 resolution using a 20× air objective (Plan Apo M27, NA 0.8; Zeiss, Jena, Germany) and 1 airy unit for the pinhole. While our previous studies quantified cytoskeletal distributions [14,15,23], they did not explicitly address the association of cytoskeletal quantity and spatial embedding.

### 4.2. Morphological Reconstruction and Editing

Digital reconstructions of neuronal morphology are lists of interconnected compartments, each representing a small section of neurites between two consecutive tracing locations [29]. Dendritic morphology was semi-automatically reconstructed and edited from the two-channel confocal images using neuTube [57]. A third channel (the pseudo membrane), created by combining microtubule and F-actin signals in FIJI [58], was used for tracing the primary reconstruction. Next, any inaccuracies in tree topology were resolved with the TREES Toolbox [34]. The resultant files were then manually curated in neuTube once more. 

Each neuron was used as input in the Vaa3D *Multichannel_Compute* plug-in tool (run in Vaa3D version 3.1) to capture signal information from both the microtubule (MT) and F-actin substructures into the final multi-signal reconstructions. The local cytoskeletal quantity (CQ) of MT and F-actin signal within a compartment was quantified using the following equation.

CQ = F ×ASI × W

Here, F is the fraction of the dendritic compartment volume occupied by the signal, ASI is the average signal intensity, and W is local dendrite thickness, which is approximated by measuring the local diameter of the compartment. All coordinates were then multiplied by the voxel size in physical units (µm) to generate the final multi-signal reconstructions. Microtubule or F-actin quantities are referred to as just microtubule or F-actin in the remaining text for simplicity. 

The integral microtubule (*IM*) of each compartment measures the microtubule quantity throughout the upstream arbor path, starting from the soma up to that compartment. IM for the (*i* + 1)th compartment is defined as:

$${IM}_{i+1}=\left({IM}_{i}\times 0.99\right)+({MQ}_{i}\times 0.01)$$

where *MQ* is the microtubule quantity; and the *i*th compartment is the parent of the (*i* + 1)th compartment. 

All multi-signal reconstructions were programmatically processed to resolve any trifurcations and overlapping points. Additionally, all the dendritic branches were locally resampled so as to make each compartment 2 µm long. 

### 4.3. Measurement and Correlation of Morphological and Cytoskeletal Properties

Analyses were conducted both at the compartment level and branch level. Here, a dendritic branch is defined as a series of consecutive compartments beginning at a tree stem or bifurcation point and ending at the next bifurcation or at a termination point. MATLAB scripts were written to quantify, for every neuron, each compartment length, orientation, angular deviation, MT and F-actin quantities, path distance from the soma, branch order, and downstream arbor lengths. More than half a million dendritic compartments from all 17 neuron groups were measured for all the parameters. Corresponding branch level parameters were computed from the values of the constituent compartments. For example, the average branch microtubule was calculated as the mean MT quantity of the compartments forming the branch. Branch straightness was defined as the ratio of branch Euclidean length (i.e., a straight line distance between branch start and end point) to branch path length (i.e., the sum of length of all compartments in the branch). The remote branch angle was measured as the angle between a branch straight line relative to the straight line of its parent branch. 

The correlation amongst the branch level parameters was calculated for each neuron group. If two variables were significantly correlated, the correlation coefficient of their binned averages was also computed. In these cases, the independent cytoskeletal parameter (e.g., microtubule quantity or F-actin quantity) values were placed into 20 bins of approximately equal sample size. The average of the dependent properties (e.g., branch remote angle or branch straightness) at each bin were then correlated (and plotted) against the bin averages of the independent cytoskeletal properties.

For each pair of ‘sibling’ branches originating from the same bifurcation point, we also measured the magnitude and direction of their deviations from the parent branch, with positive and negative signs assigned to counterclockwise and clockwise deviations, respectively. We define branch tilt as the angle created between the parent branch and the straight line that splits the two sibling branches through the middle, creating two angles of the same magnitude.

### 4.4. Model of Dendritic Arbor Generation

The full arbor generation model is built at the single compartment resolution level and extends our previous simulation of dendrogram topology [19]. At each iteration, the model reads the compartmental composition of microtubule and F-actin as input, and outputs the developmental event (extend, bifurcate, or terminate) as well as the cytoskeletal composition and the angular deviation of the newly created child compartment(s), if any. As in our earlier model [19], the process relies on three two-dimensional sampling grids, where the dimensions are local microtubule and F-actin quantities: event grid, extension grid, and branching grid. The event grid determines the probability of extending, bifurcating, or terminating based on the compartment MT and F-actin composition. Similarly, the extension and branching grids determine the cytoskeletal composition and angular deviation of the newly created child compartment(s) also based on the (parent) compartment microtubule and F-actin quantities. Within each sampling grid bin, the model choses the topological event, cytoskeletal composition, and angular orientation that best match the integral microtubule of the parent compartment. 

### 4.5. Comparison of Real and Simulated Arbor Properties

Once virtual neuron arbors are generated, they are statistically compared against their real counterparts. First, the L-Measure [59] morphometric tool is used to quantify 10 distinct properties for each real and simulated neuron. These 10 measures are then separately compared between real and simulated neuron groups using the Student’s *t*-test (if *N* > 10 neurons per group) or the non-parametric Wilcoxon Rank Sum test (if *N* ≤ 10 per group). 

Persistent vector analysis was carried out using a publicly available automated tool [60], which automatically produce one persistent vector file for each neuronal reconstruction file. A new analysis script measured the arccosine distances between the persistence vectors of each neuron pair to compare within-group differences against between-group differences. 

Dendritic density analysis was carried out using TREES Toolbox [34]. Two-dimensional density matrices were created since the sensory da neurons lie flat on the cuticle of the *Drosophila* larva. These matrices were then vectorized and analyzed in terms of arccosine distances as described above for the persistence vectors.

## Figures and Tables

**Table 1:** Abbreviation, neuron class, subtype, genotype, morphological characteristics, number of neurons, and data sources information for the 17 neuron groups used in this study.

Abbreviation	Neuron Class	Neuron Subtype	Genotype	Morphology Description	Number of Neurons	Source Publication
C1-ddaE-WT	Class I	ddaE	Wild type	Simple dendritic arbors, secondary arbors tend to extend towards one direction	19	[15]
C1-vpda-WT	Class I	vpda	Wild type	Simple dendritic arbors, secondary arbors tend to extend towards both direction	8	[15]
C1-vpda-mts-IR	Class I	vpda	mts-Knockdown	Increased terminal complexity	9	[15]
C1-vpda-mts-OE	Class I	vpda	mts-Overexpression	Reduced size, and complexity	12	[15]
C4_WT	Class IV	ddaC	Wild type	Large complex dendritic arbors	10	[15]
C4_bdwf-IR	Class IV	ddaC	CG3995/bdwf Knockdown	Reduced size and complexity	8	[30]
C4_form3-OE	Class IV	ddaC	Formin 3 Overexpression	Reduced size	9	[23]
C4_mts-IR	Class IV	ddaC	mts-Knockdown	Reduced size	11	[15]
C4_mts-OE	Class IV	ddaC	mts Overexpression	Reduced size	10	[15]
C4_cut_IR	Class IV	ddaC	cut Knockdown	Reduced size and terminal complexity	8	[30]
C4_RpL4-IR	Class IV	ddaC	RpL4-Knockdown	Reduced size and complexity	10	[30]
C4_RpL17-IR	Class IV	ddaC	RpL17-Knockdown	Reduced size and complexity	6	[31]
C4_RpL22-IR	Class IV	ddaC	RpL22-Knockdown	Reduced size and complexity	10	[30]
C4_RpL31-IR	Class IV	ddaC	RpL31-Knockdown	Reduced size and complexity	9	[30]
C4_RpL35A-IR	Class IV	ddaC	RpL35A-Knockdown	Reduced size and complexity	13	[31]
C4_RpS10b-IR	Class IV	ddaC	RpS10b-Knockdown	Reduced size and complexity	8	[31]
C4_RpS24-IR	Class IV	ddaC	RpS24-Knockdown	Reduced size and complexity	7	[30]

**Table 2:** Pearson’s correlations between average branch MT, F-actin or integral MT and remote branch angle or branch straightness for all 17 neuron groups. Values not reaching statistical significance after False Discovery Rate correction for multiple testing were substituted with 0 s.

Neuron Groups	MT vs. Remote Angle	F-Act vs. Remote Angle	Integral MT vs. Remote Angle	MT vs. Branch Straightness	F-Act vs. Branch Straightness	Integral MT vs. Branch Straightness
C1-ddaE-WT	−0.80	−0.68	0	0.64	0	0.64
C1-vpda-WT	−0.40	0	0	0	0	0
C1-vpda-mts-IR	−0.58	0	0	0	0	0
C1-vpda-mts-OE	−0.23	0	0	0	0	0
C4_WT	−0.82	−0.88	−0.51	0.72	0.84	0.11
C4_bdwf-IR	−0.73	0	0	0.69	0.68	0
C4_form3-OE	−0.71	−0.88	−0.67	0.66	0.86	0.83
C4_mts-IR	−0.72	−0.63	0	0	0.89	0
C4_mts-OE	−0.88	0	0	0.82	0.61	0.46
C4_cut_IR	−0.75	0.80	0	0.85	0	0.35
C4_RpL4-IR	−0.72	0	0	0.63	0.74	0.53
C4_RpL17-IR	−0.62	0	−0.33	0.72	0.72	0.33
C4_RpL22-IR	−0.14	0	−0.38	0.00	0.53	0
C4_RpL31-IR	−0.62	0	0	0.62	0	0.50
C4_RpL35A-IR	−0.62	0	−0.54	0.67	0.48	0.66
C4_RpS10b-IR	−0.59	−0.15	0	0.68	0.75	0.49
C4_RpS24-IR	−0.80	−0.14	−0.46	0.68	0.55	0.30

**Table 3:** False Discovery Rate-corrected *t*-test or Wilcoxon test *p* values of comparisons for 10 L-Measure morphometrics between real and simulated neuron across 17 cell groups.

Neuron Groups	Number of Branches	Height	Width	Length	Max Path Distance	Max Euclidean Distance	Max Branch Order	Contraction	Partition Asymmetry	Local Bifurcation Angle
C1-ddaE-WT	1	1	0.99	1	1	1	1	0.99	1	1
C1-vpda-WT	1	0.98	1	0.96	0.90	0.93	1	0.90	1	0.96
C1-vpda-mts-IR	1	1	1	1	0.98	0.97	1	1	1	0.93
C1-vpda-mts-OE	0.94	0.98	0.77	0.88	0.92	0.79	0.79	0.95	0.68	0.95
C4_WT	1	0.97	0.85	1	0.94	0.76	0.79	0.73	0.82	0.91
C4_bdwf-IR	1	0.90	0.94	0.94	0.90	0.86	0.90	0.99	0.66	1
C4_form3-OE	1	0.65	0.53	0.92	0.95	0.62	0.96	1	0.85	0.93
C4_mts-IR	1	0.98	0.96	1	0.99	1	0.74	0.98	0.91	0.97
C4_mts-OE	1	0.68	0.94	1	0.97	0.85	0.73	1	1	0.85
C4_cut_IR	1	0.82	0.90	0.78	0.98	0.93	0.94	0.96	0.94	0.99
C4_RpL4-IR	1	0.97	1	1	0.97	0.97	1	1	1	1
C4_RpL17-IR	1	0.66	0.85	0.92	0.97	1	1	0.94	0.91	1
C4_RpL22-IR	1	1	1	1	1	1	1	0.91	0.68	0.94
C4_RpL31-IR	1	0.97	0.98	0.95	0.88	0.98	0.96	0.95	1	0.93
C4_RpL35A-IR	1	1	1	1	1	1	1	1	1	0.95
C4_RpS10b-IR	1	1	0.98	0.96	1	0.93	1	0.94	0.96	0.96
C4_RpS24-IR	0.96	0.97	0.83	0.92	0.64	0.97	0.58	0.93	0.78	0.80

**Table 4:** False Discovery Rate-corrected *t*-test *p* values of comparisons for pairwise distances in persistence vectors or global density vectors between within-condition (real vs. real and simulated vs. simulated) and across-conditions (real vs. simulated) for all 17 cell groups. Values greater than 0.1 mean failure to reject the null hypothesis that the real and simulated neurons belong to the same distribution.

Neuron Group	Persistence Vectors	Arbor Density
C1-ddaE-WT	0.92	1
C1-vpda-WT	0.93	0.99
C1-vpda-mts-IR	0.93	0.99
C1-vpda-mts-OE	0.91	1
C4_WT	0.99	0.99
C4_bdwf-IR	0.94	1
C4_form3-OE	0.82	0.99
C4_mts-IR	0.88	1
C4_mts-OE	0.84	1
C4_cut_IR	0.91	1
C4_RpL4-IR	0.99	1
C4_RpL17-IR	0.72	0.99
C4_RpL22-IR	0.85	1
C4_RpL31-IR	0.82	1
C4_RpL35A-IR	0.87	1
C4_RpS10b-IR	0.98	1
C4_RpS24-IR	0.98	0.99

## Data Availability

All reconstructions were submitted to NeuroMorpho.Org and will be available in the Cox and Ascoli archives upon publication. The processed data were deposited in Mendeley.com along with the analysis and modeling code for open-access release upon publication of this manuscript (DOI: 10.17632/cj69j8bpn8.1).

## Supplementary Materials

The supporting information can be downloaded at: https://www.mdpi.com/article/10.3390/ijms24076741/s1.
