# Supplementary material for: Local Microtubule and F-Actin Distributions Fully Constrain the Spatial Geometry of Drosophila Sensory Dendritic Arbors
Source: Int J Mol Sci. 2023 Apr 4;24(7):6741. doi: 10.3390/ijms24076741 (PMC10095360; doi:10.3390/ijms24076741)
Supplement: Supplementary file 1 [file ijms-24-06741-s001.zip › ijms-2277650-supplementary/Supplementary_Figures_and_Tables/Supplementary_Figures_Tables_Legends.docx]

## Supplementary Figures

**SF1:** Overall morphology along with cytoskeletal distribution of the simulated neurons. Single representative neurons from (A) 4 Class I neuron types (1 WT subclasses and 2 mutant groups) and (B) 8 Class IV neuron types (1 WT ddaC and 7 mutant groups) are shown, each in two separate images displaying microtubule (red hue) and F-actin (green-blue hue) distributions, respectively. Color bars are constant for all 17 neurons groups. All Scale bars represent 100 µm.

**SF2:** Density profile comparison between real and simulated neuron groups. Arbor density averaged across all neurons from each group for the remaining 7 Class IV groups (RpL4-IR, RpL17-IR, RpL22-IR, RpL31-IR, RpL35A-IR, RpS10b-IR and RpS24-IR). Heatmaps for both real (left) and simulated (right) neurons are provided for each group.

## Supplementary Tables

ST1: Correlation between branch order and branch angle, average internal branch length, and average terminal branch length for real as well as simulated neurons from all 17 neuron groups.

ST2: Correlations between local and remote branch angle, between the angular deviations of sibling branch pairs (D1 and D2), and between maximal angle and branch tilt for all 17 neuron groups.
