# Supplementary figures and images for: Local Microtubule and F-Actin Distributions Fully Constrain the Spatial Geometry of Drosophila Sensory Dendritic Arbors
Source: Int J Mol Sci. 2023 Apr 4;24(7):6741. doi: 10.3390/ijms24076741 (PMC10095360; doi:10.3390/ijms24076741)

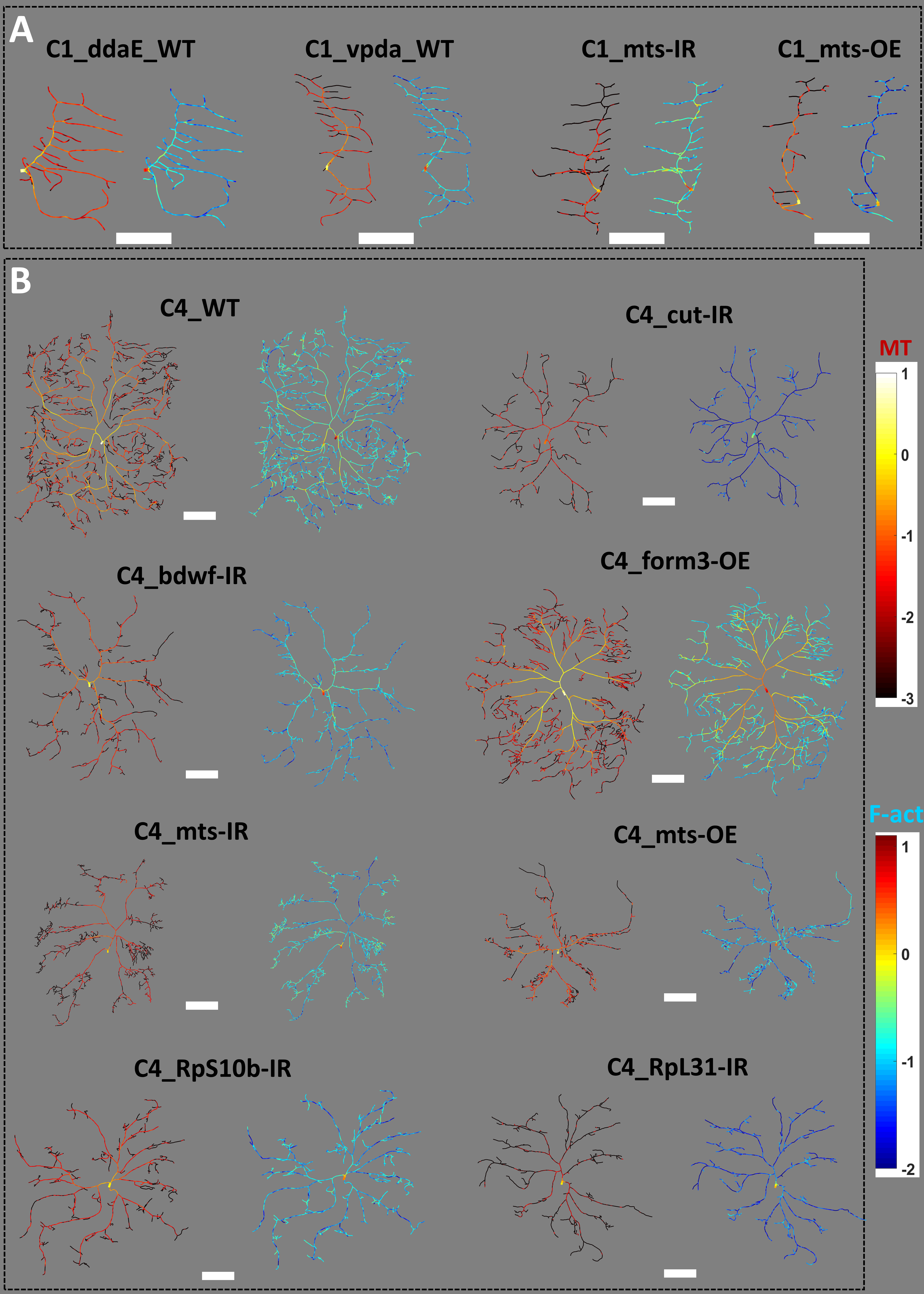

Supplement: Supplementary file 1 [file ijms-24-06741-s001.zip › ijms-2277650-supplementary/Supplementary_Figures_and_Tables/SF1.TIF]

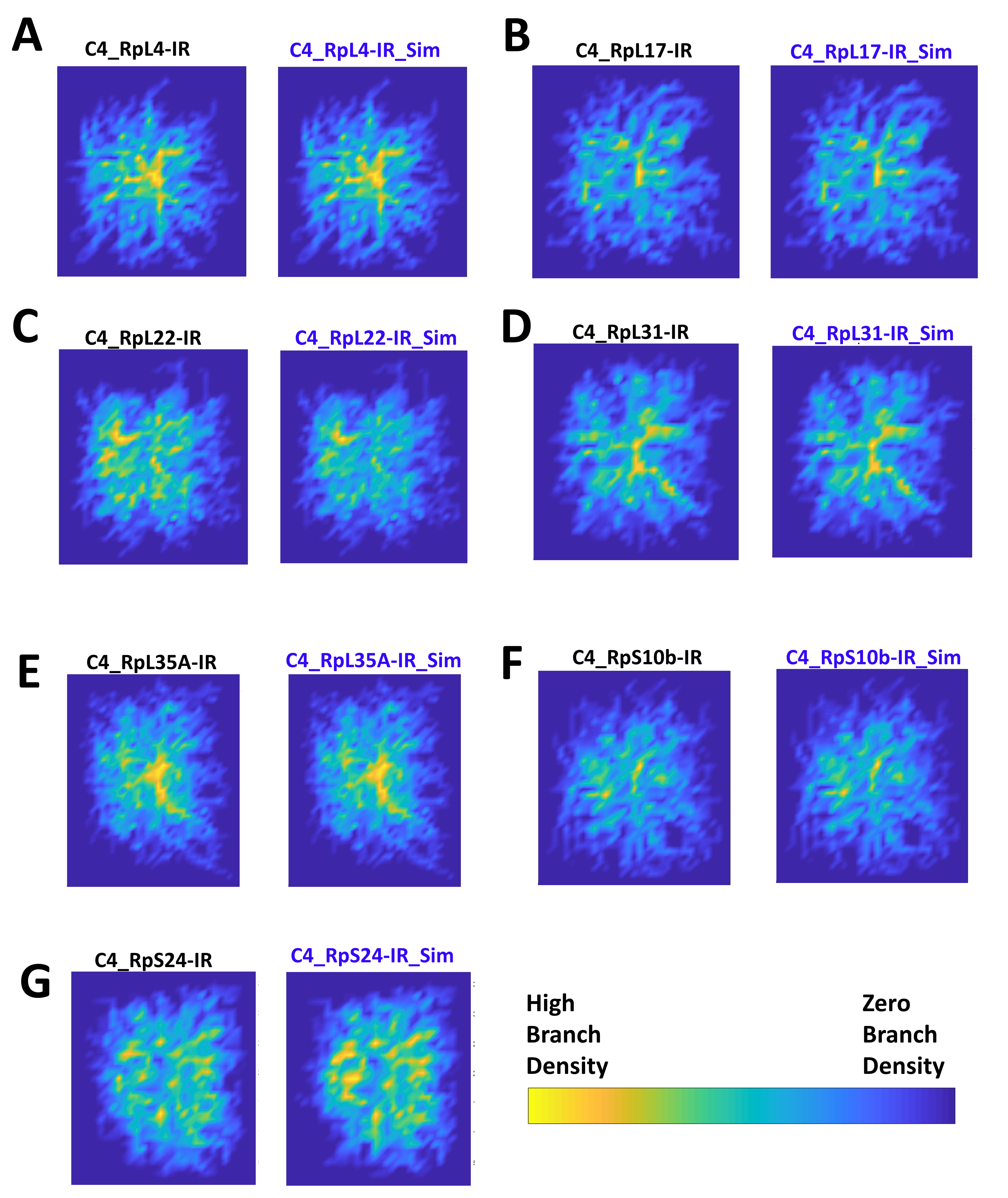

Supplement: Supplementary file 1 [file ijms-24-06741-s001.zip › ijms-2277650-supplementary/Supplementary_Figures_and_Tables/SF2.TIF]
